# Chlorophyll Fluorescence Imaging as a Tool for Evaluating Disease Resistance of Common Bean Lines in the Western Amazon Region of Colombia

**DOI:** 10.3390/plants11101371

**Published:** 2022-05-21

**Authors:** Juan Carlos Suárez, José Iván Vanegas, Amara Tatiana Contreras, José Alexander Anzola, Milan O. Urban, Stephen E. Beebe, Idupulapati M. Rao

**Affiliations:** 1 Programa de Ingeniería Agroecológica, Facultad de Ingeniería, Universidad de la Amazonia, Florencia 180001, Colombia; vanegas.agroeco@gmail.com (J.I.V.); 1993anzola@gmail.com (A.T.C.); amaratatis18@gmail.com (J.A.A.); 2 Centro de Investigaciones Amazónicas (CIMAZ)—Macagual César Augusto Estrada González, Grupo de Investigaciones Agroecosistemas y Conservación en Bosques Amazónicos-GAIA, Florencia 180001, Colombia; 3 International Center for Tropical Agriculture (CIAT), Km 17 Recta Cali-Palmira, Cali 763537, Colombia; m.urban@cgiar.org (M.O.U.); s.beebe@cgiar.org (S.E.B.); i.rao@cgiar.org (I.M.R.)

**Keywords:** acid soil, agronomic performance, disease susceptibility index, dry matter accumulation, grain yield, high temperature, photochemical processes, photosynthetic response

## Abstract

The evaluation of disease resistance is considered an important aspect of phenotyping for crop improvement. Identification of advanced lines of the common bean with disease resistance contributes to improved grain yields. This study aimed to determine the response of the photosynthetic apparatus to natural pathogen infection by using chlorophyll (Chl_a_) fluorescence parameters and their relationship to the agronomic performance of 59 common bean lines and comparing the photosynthetic responses of naturally infected vs. healthy leaves. The study was conducted over two seasons under acid soil and high temperature conditions in the western Amazon region of Colombia. A disease susceptibility index (DSI) was developed and validated using chlorophyll a (Chl_a_) fluorescence as a tool to identify Mesoamerican and Andean lines of common bean (*Phaseolus vulgaris* L.) that are resistant to pathogens. A negative effect on the functional status of the photosynthetic apparatus was found with the presence of pathogen infection, a situation that allowed the identification of four typologies based on the DSI values ((i) moderately resistant; (ii) moderately susceptible; (iii) susceptible; and (iv) highly susceptible). Moderately resistant lines, five of them from the Mesoamerican gene pool (ALB 350, SMC 200, BFS 10, SER 16, SMN 27) and one from the Andean gene pool (DAB 295), allocated a higher proportion of energy to photochemical processes, which increased the rate of electron transfer resulting in a lower sensitivity to disease stress. This photosynthetic response was associated with lower values of DSI, which translated into an increase in the accumulation of dry matter accumulation in different plant organs (leaves, stem, pods and roots). Thus, DSI values based on chlorophyll fluorescence response to pathogen infection could serve as a phenotyping tool for evaluating advanced common bean lines. Six common bean lines (ALB 350, BFS 10, DAB 295, SER 16, SMC 200 and SMN 27) were identified as less sensitive to disease stress under field conditions in the western Amazon region of Colombia, and these could serve as useful parents for improving the common bean for multiple stress resistance.

## 1. Introduction

The common bean (*Phaseolus vulgaris* L.) is the most important food legume and it is particularly valued for its protein and micronutrient content [1]. In parts of Africa and Latin America, it provides an average of 15% of total daily calories and 36% of daily protein content [2]. Most of the production of common bean is carried out by small farmers under adverse climatic conditions in the tropics and subtropics [3,4]. The growing conditions in these regions, particularly in the humid tropics, favor numerous infectious diseases caused by fungi, viruses, bacteria and nematodes that result in yield losses up to 100% [5,6,7].

Common bean germplasm is divided into two major gene pools that have been individually domesticated: the Andean gene pool, mainly large-seeded, and the Mesoamerican gene pool, which is small- to medium-seeded [8]. The infection-causing pathogens (fungi, viruses, bacteria and nematodes) are also classified into two genetic groups (Andean and Mesoamerican), which co-evolved with the gene pools of their host [9]. While the Andean group of pathogens affect Andean beans, Mesoamerican pathogens are more pathogenic to Mesoamerican beans, as well as, to some extent, Andean beans, thus showing a greater diversity of virulence [6]. Biotic stress caused by pathogens in plants inevitably induces various changes in their physiological functions, resulting in metabolic disorders due to infections or nutritional deficiencies [10]. Biotic stress symptoms can generally be seen in leaves, stems and roots, but lesions can also appear in seeds, leading to losses in productivity and grain quality [5]. Thus, accurate assessment of plant symptoms can be used as a proxy indicator when monitoring diseases, estimating yield loss and developing resistant genotypes against plant diseases [11].

In breeding programs, the evaluation of disease resistance or biotic stress resistance is based on disease severity, which is defined as the area of plant tissue infected by disease-causing organisms and expressed as a percentage of the total amount of plant tissue [7,12]. Likewise, evaluation relies heavily on traditional methods such as rating scales based on visible symptoms [13], which in most cases measure severity based on rater subjectivity that often lacks precision, reproducibility and traceability [14]. Plants have evolved complex sensory mechanisms to identify biotic invasion and overcome detriments to growth, yield and survival [15]. Changes induced under both light and dark periods are critical for plant survival [16]. Disease resistance has been observed to have a positive influence on plant photosynthesis compared to sensitive cultivars [17]. A disease-resistant cultivar usually shows higher rates of photosynthesis, electron transport and dark respiration, eventually reaching high yields in regions where widespread diseases are present [17,18]. However, the presence of a disease in a susceptible plant triggers alterations in morphology, physiological functions and plant integrity that can cause partial or total damage [10], as these are determinants in the plant’s ability to adapt to specific environmental conditions [19].

Among different stress responses, photosynthesis plays an important role in plant disease defense responses [20]. The reduction of photosynthesis by the pathogen can be caused by the deterioration of the photosynthetic apparatus. This can substantially reduce the ability of the leaves to fix CO_2,_ that is necessary for the formation of the photosynthates that are required during the reproductive phase [21]. In addition, stress induced by diseases can increase the excitation energy, to the point that it exceeds the amount necessary for photosynthetic metabolism, generating reactive oxygen species (ROS) [22] as well as a series of related changes in chloroplast–disease interaction [23]. These include: (i) fluctuation of chlorophyll fluorescence and reduced chlorophyll pigmentation [24,25,26], (ii) inhibition of photosystem efficiency [27], (iii) unbalanced accumulation of photoassimilates [28], (iv) changes in chloroplast structure and function [29] and (v) decreases in F_v_/F_m_, ΦPSII and increases in NPQ heat energy dissipation [30], and these changes can alter leaf temperature. For example, when pathogens directly or indirectly impair transpiration, the net effect is a lack of thermal regulation resulting in an increase in leaf temperature [19,31].

Improving disease resistance by developing and identifying more resistant genotypes is considered the most sustainable method to reduce yield losses due to disease [6,7]. This disease resistance breeding approach is an efficient, reliable and inexpensive way to sustainably manage disease with fewer chemical inputs [32]. Among plant responses to stress, chlorophyll fluorescence is very sensitive to plant physiological changes and is used to measure the physiological state of a plant under both biotic [13,25,26,33] and abiotic [34,35] stress conditions. Chlorophyll a (Chl_a_) fluorescence results from light energy absorbed by chlorophyll in photosystem II (PSII), which can be used for photosynthesis (qP, photochemical quenching), re-emitted as fluorescence or lost as heat (NPQ, non-photochemical quenching). If the quantum yield of one of the processes decreases, the quantum yield of one or both of the other two processes will increase [13,36]. One of the main strengths of Chl_a_ fluorescence measurements is that many stresses can be detected before any sign of visible damage occurs [37,38], depending on the type of stress suffered by the plant, so there is great potential for helping to minimize crop production problems through early detection of stress factors [39].

The objective of this work was to determine the response of the photosynthetic apparatus to natural pathogen infection by using Chl_a_ fluorescence parameters in different common bean lines and comparing the response from naturally infected vs. healthy leaves under field conditions. Based on this information on chlorophyll fluorescence imaging, a disease susceptibility index (DSI) was developed for phenotyping different bean lines and to identify the less susceptible ones under the field conditions (acid soils and high temperature) of the Colombian Amazon. For this purpose, the existence of variability in the tolerance-adjustment mechanism of the photosynthetic apparatus to disease stress was hypothesized. To test this, a DSI was developed using chlorophyll fluorescence as a tool to evaluate the disease resistance of the Mesoamerican and the Andean lines of common bean and identify sensitive genotypes. The DSI was mostly based on the comparison of light use and paths of energy transformation (i.e., energy that passes to photochemistry ΦII, dissipates as heat ΦNPQ and dissipates in a non-regulated manner ΦNO) in healthy and naturally infected leaves.

## 2. Results

### 2.1. Response of the Photosynthetic Apparatus to Disease Stress in Leaves of Phaseolus vulgaris

**Figure 1:**
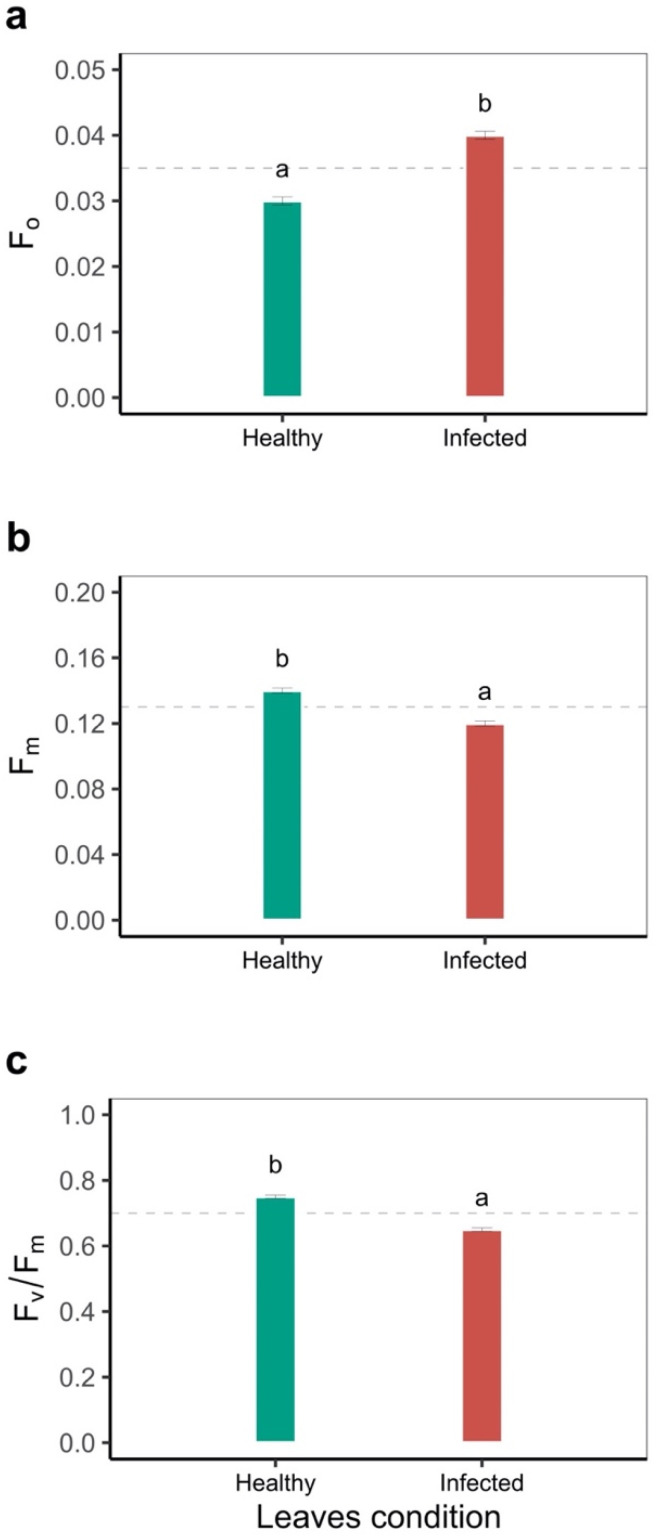
Photochemical status of fully oxidized and reduced photochemistry II (PSII) of different bean genotypes with susceptibility to disease stress. (**a**) Initial fluorescence (F_0_); (**b**) maximal fluorescence (F_m_); (**c**) the maximum quantum yield of the PSII photochemistry F_v_/F_m_. The dashed black line corresponds to the mean of each variable. a, b: Letters for the bars for each leaf condition indicate statistically significant differences based on the LSD means test (*p* < 0.05). Results include the mean ± SE (n = 2832 leaves).

**Figure 2:**
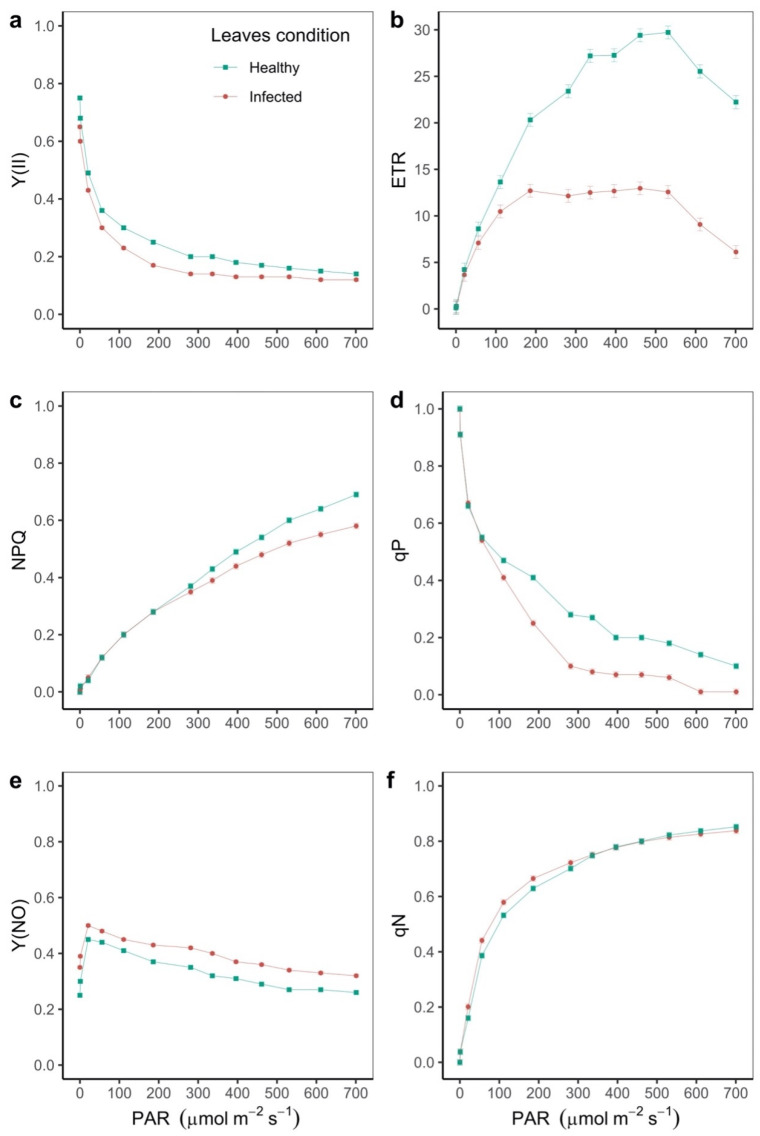
Response of the photosynthetic apparatus to disease stress. (**a**) The effective quantum yield of photochemical energy conversion in PSII (Y(II)); (**b**) non-photochemical quenching (NPQ); (**c**) the quantum yield of non-regulated energy dissipation (Y(NO)); (**d**) the apparent electron transport rate (ETR); (**e**) the coefficient of photochemical quenching (qP); (**f**) the non-photochemical quenching coefficient (qN). PAR: photosynthetically active radiation. Each data point on the curve represents the mean and standard deviation (n = 708 plants for each leaf condition).

Chlorophyll fluorescence parameters showed that the initial fluorescence yield (F_o_) was 38.7% higher in infected leaves, contrary to what was presented for the maximum fluorescence (F_m_), which was 25% higher in healthy leaves (*p* < 0.05). The maximum quantum yield of the PSII photochemistry (F_v_/F_m_) for both healthy and infected leaf conditions was found to be below 0.82, which suggests that stress conditions exist due to acid soil and high temperature stress (Figure 1). When analyzing the increase of the irradiance level, we found that the effective PSII quantum yield (Y(II)) was significantly reduced for both conditions analyzed. The value was always higher in the healthy leaf, where at 300 μmol m^−2^ s^−1^ the value of Y(II) was 0.20 for healthy and 0.14 for infected leaves, respectively (Figure 2a). As for the fraction of the energy dissipated as heat related to non-photochemical quenching (NPQ), it was found that in the two conditions with increasing PAR, the NPQ also increased. The curves were different from 300 μmol m^−2^ s^−1^, reaching a difference of 14.2% at 700 μmol m^−2^ s^−1^ between the two conditions (*p* < 0.05, Figure 2b). For the quantum yield of non-regulated energy dissipation (Y(NO)), we found that it increased up to 21 μmol m^−2^ s^−1^, at which point the values were 0.45 and 0.50 for the healthy and infected leaves, respectively, and reached a difference of 23% between the two leaf conditions at 700 μmol m^−2^ s^−1^ of PAR (*p* < 0.05, Figure 2c). The apparent electron transport rate (ETR) was the most sensitive variable. The greatest difference was observed between the two conditions at PAR 500 μmol m^−2^ s^−1^, where the ETR in the healthy leaf was 3.6 times higher compared to the infected leaves (*p* < 0.05, Figure 2d). The coefficient of photochemical quenching (qP) decreased with increasing PAR, with the qP value being higher in the healthy leaf (*p* < 0.05, Figure 2e). Finally, the non-photochemical quenching coefficient (qN) was proportional to PAR, with no differences from PAR 350 μmol m^−2^ s^−1^ (*p* < 0.05, Figure 2f).

**Figure 3:**
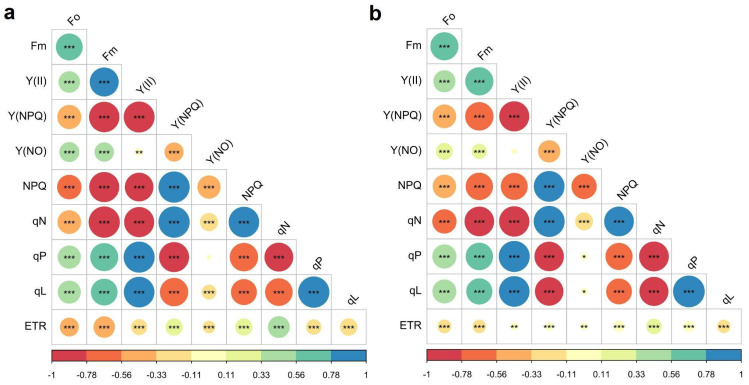
Relationships between different variables of chlorophyll fluorescence measured in *Phaseolus vulgaris* leaves. (**a**) Healthy leaves; (**b**) infected leaves. Maximal fluorescence (F_m_), the effective quantum yield of photochemical energy conversion in PSII (Y(II)), quantum yield of regulated energy dissipation Y(NPQ), quantum yield of nonregulated energy dissipation Y(NO), non-photochemical quenching (NPQ), the non-photochemical quenching coefficient (qN), the coefficient of photochemical quenching (qP), coefficient of photochemical quenching (qL), and the apparent electron transport rate (ETR).

When analyzing the relationships between the different variables of chlorophyll fluorescence in both healthy and infected leaves, contrasting differences were found (Figure 3). For example, in healthy leaves all correlations were significant, excluding the relationship between qP and Y(NO). In infected leaves, no correlation was found between Y(NO) and Y(II). Among the positive correlations (green to blue gradient), we found a reduction in the correlation between qN and ETR; while in the healthy leaf it had a relationship of 0.45, it decreased to 0.22 in the infected leaf (*p* < 0.05, Figure 3). A similar situation occurred regarding the correlation of ETR with NPQ and Y(NPQ); under the healthy conditions, the relationship was 0.28, but the infected leaf presented a correlation of 0.11 and 0.06, respectively (*p* < 0.05, Figure 3). In general, the variable with the greatest variation between the two conditions was the electron transfer rate (ETR) (*p* < 0.05, Figure 3).

### 2.2. Phenotyping of Advanced Lines of Phaseolus vulgaris for Susceptibility to Disease Stress

**Figure 4:**
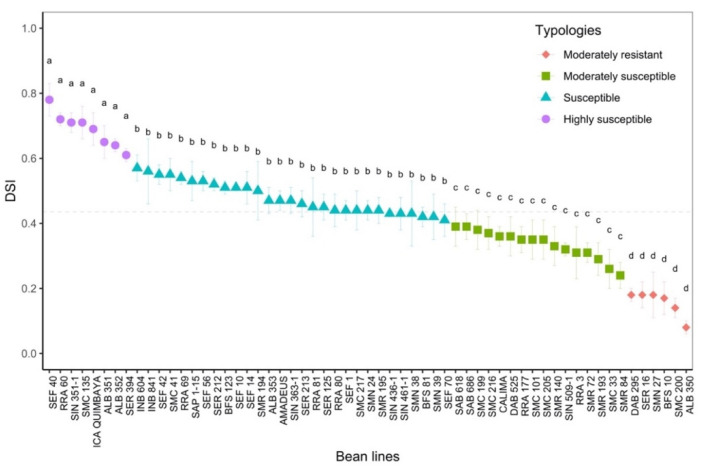
Disease susceptibility index (DSI) of advanced bean lines. The dashed black line corresponds to the mean of each variable. a, b, c, d: Letters at each point for each common bean forward line indicate statistically significant differences based on the LSD means test (*p* < 0.05). Results include the mean ± SE (n = 708).

Using chlorophyll fluorescence imaging, the disease susceptibility index (DSI) was designed to phenotype advanced bean lines. When we analyzed the response of the photosynthetic apparatus using DSI, we found contrasting differences (*p* < 0.05, Figure 4, Table 1. From the cluster analysis, four DSI-related typologies were obtained (moderately resistant, moderately susceptible, susceptible and highly susceptible).

- *Moderately resistant* (MR; n = 6; 21.8% of the total genotypes evaluated) was a typology characterized by low and high levels of F_o_ and F_m_, respectively, in the dark adaptation phase. At the level of energy distribution in the different fractions, this typology assigned the highest proportion to qP (*p* < 0.05) and, likewise, to efficiency in the electron transfer rate (ETR, *p* < 0.05). This typology had a lower DSI with an overall average of 0.16 ± 0.02 (represented by lines ALB 350, SMC 200, BFS 10, DAB 295 and SER 16). In terms of agronomic response (Table 1), these lines accumulated a greater amount of biomass in different organs, such as the root, stem, leaves and canopy biomass (*p* < 0.001), and showed a greater number of pods (4.6 ± 0.29 g). The plants accumulated canopy biomass twice as much compared to the highly susceptible type.
- *Moderately susceptible* (MS; DSI = 0.33, n = 16; 27.1%) was a typology that was characterized by an increase in the fraction of non-regulated energy (qN), and its reaction centers were moderately reduced so that its electron transfer rate (ETR) was lower compared to the moderately resistant bean lines. The best and lowest rated lines in terms of DSI were SMR 84 and SAB 686, respectively. This typology reduced its pod biomass; however, a strong presence of flowers was found with an increase in root biomass.
- *Susceptible* (S; DSI = 0.48, n = 29; 49.1%) was a typology where Y(II) was reduced by 40% and the fraction of energy dissipated as heat (NPQ) and non-regulated (qN) was increased by 46% and 40%, respectively, in relation to the moderately resistant bean lines. Likewise, this typology showed a considerable reduction in the fraction of energy distributed to the photosynthetic process (qP, 44.8%) and in the electron transport rate (ETR, 53.2%). The lines that conformed to this typology experienced a greater effect from disease stress on pod production, as well as on the other plant organs.
- *Highly susceptible* (HS; DSI = 0.69, n = 8; 13.5%) was a typology where the energy was distributed in a higher proportion in the form of heat dissipation (NPQ) and as non-regulated (qN), and the efficiency of the photosynthetic apparatus was very low (qP and ETR). At the agronomic level, a significant effect was found since several plant organs did not show adequate development; however, an increase in root biomass was found at the expense of shoot growth.

**Figure 5:**
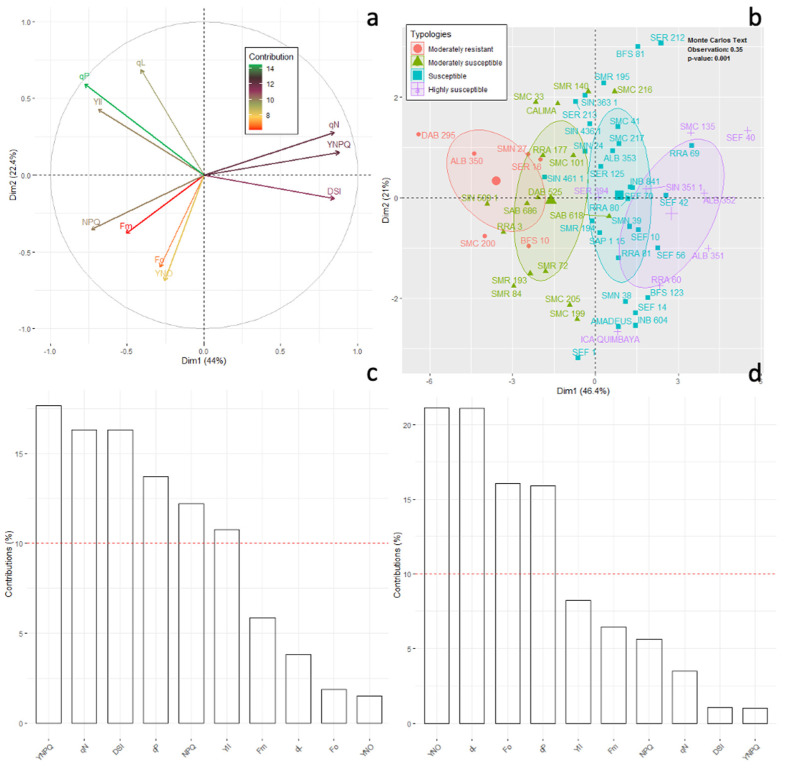
Projection of chlorophyll fluorescence variables grouped by the different types of common bean genotypes on the F1/F2 factorial plane of the principal component analysis (PCA). (**a**) Correlation circle for chlorophyll fluorescence variables. The variables with the highest to the lowest contribution are denoted with a color gradient from green to red. (**b**) Classification of common bean genotypes from chlorophyll fluorescence variables and disease susceptibility index (DSI). (**c**,**d**) Contribution of chlorophyll fluorescence variables to the formation of the F1/F2 principal components of the PCA under different types of common bean genotypes. Variables above the red line presented higher contributions in each component (*p* < 0.05). Acronyms are shown in Table 1.

The PCA of chlorophyll fluorescence and DSI variables could explain 67.4% of the total variability along the first two axes, separating the different bean lineage typologies (*p* < 0.05) (Figure 5a). Axis 1 (46.4%) contrasted bean lines (highly susceptible) with higher fractions of energy dissipated as heat (NPQ and Y(NPQ)) and lines with higher DSI, qP and ETR values (moderately resistant) (Figure 5a). Axis 2 (21%) was notable for its high F_o_ value, associated with the susceptible and highly susceptible bean lines (Figure 5a,b). A Monte-Carlo test showed that the difference in chlorophyll fluorescence values significantly (*p* < 0.001) differentiated the different typologies of the bean lines in our study, with an explained variability of 36% (Figure 5b). When we analyzed the major contributing variables in both 1 and 2 in the PCA, we found that those above the red line in Figure 5c,d were statistically the most important in separating the bean lines in each of the typologies according to the level of disease susceptibility.

### 2.3. Fluorescence and Images of Chlorophyll A (Chl_a_) Fluorescence Parameters for Phenotyping of Advanced Lines of Phaseolus vulgaris

**Figure 6:**
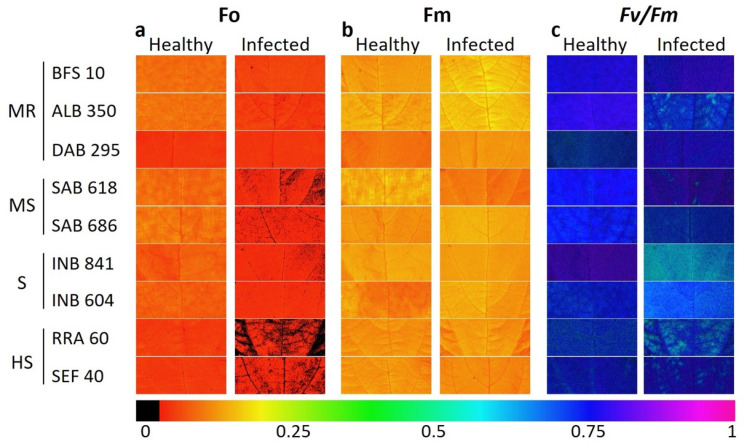
Images of chlorophyll fluorescence parameters of different advanced lines of common beans under two leaf conditions (healthy and infected). BSF 10, ALB 350, DAB 295: moderately resistant (MR). SAB 618, SAB 686: moderately susceptible (MS). INB 841 and INB 604: susceptible (S). RRA 60 and SEF 40: highly susceptible (HS). (**a**) Initial fluorescence (F_o_); (**b**) maximum fluorescence (F_m_); (**c**) maximum quantum efficiency of PSII (F_v_/F_m_). The change in the color gradient from violet to red signifies a change in value from higher to lower for each variable.

The bean lines with the highest DSI values from all typology groups were obtained from quantitative analysis and from Chl_a_ fluorescence parameters. For example, in the dark adaptation phase, we found red color gradients in the leaves with disease stress for the variable F_0_ (Figure 6a), up to the point of having black areas caused by pathogen necrosis in some bean lines of the highly susceptible typology such as RRA 60 and SEF 40 (Figure 6b). The maximum quantum yield for the PSII photochemistry (F_v_/F_m_, Figure 6c) was found in the healthy leaves for the lines BFS 10, ALB 350 and DAB 295, which corresponded to the moderately resistant typology. When decreasing the level of resistance in the bean lines of the other typologies, the value F_v_/F_m_ was significantly reduced in the infected leaf, a condition that can be observed in the gradient changing from violet and blue to light green (Figure 6c).

**Figure 7:**
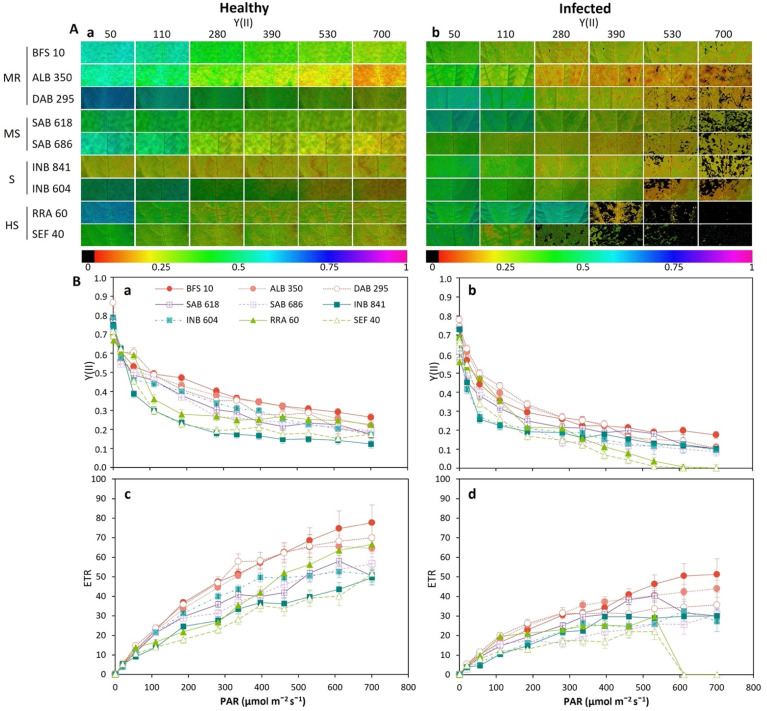
Response to disease stress as a function of the photosynthetically active radiation (PAR) levels of common bean lines conforming to different typologies. BSF 10, ALB 350, DAB 295: moderately resistant (MR). SAB 618, SAB 686: moderately susceptible (MS). INB 841 and INB 604: susceptible (S). RRA 60 and SEF 40: highly susceptible (HS). (**A**) Images of the parameter Y(II) obtained by IMAGINE-PAM; the change in the color gradient from violet to red signifies a change in value from higher to lower for each variable. (**B**) Fast light curves of Y(II) as a function of PAR: (**Ba**) Y(II) in healthy leaves; (**Bb**) Y(II) in infected leaves; (**Bc**) ETR in healthy leaves; (**Bd**) ETR in infected leaves. Means and error bars correspond to 96 AOIs (areas of interest) for each condition (healthy and infected); i.e., six AOIs were taken for each leaf, among four plant leaves selected between the fifth and eighth trifoliate leaves (from apex to base) at the beginning of physiological maturity, from four plants of each advanced line of common bean.

We found that Y(II) decreased with increasing irradiance level (PAR), being different in both the different leaf conditions (healthy and infected) and among the different advanced lines of common beans (Figure 7). The line BFS 10 showed in both healthy and infected leaves a higher level of Y(II) (*p* < 0.05, Figure 7A). Highly susceptible lines such as SEF 40, RRA 60 and INB 604 showed zero Y(II) in the infected leaves from 280 μmol m^−2^ s^−1^ of PAR (Figure 7(Ab)). It is worth noting that these susceptible lines were of interspecific origin. The ETR was reduced by an average of 34% in the infected leaves for all the bean lines evaluated, reaching the point of having zero values at 600 μmol m^−2^ s^−1^ of PAR for the SEF 40 and RRA 60 lines (Figure 7B(a,b)).

**Figure 8:**
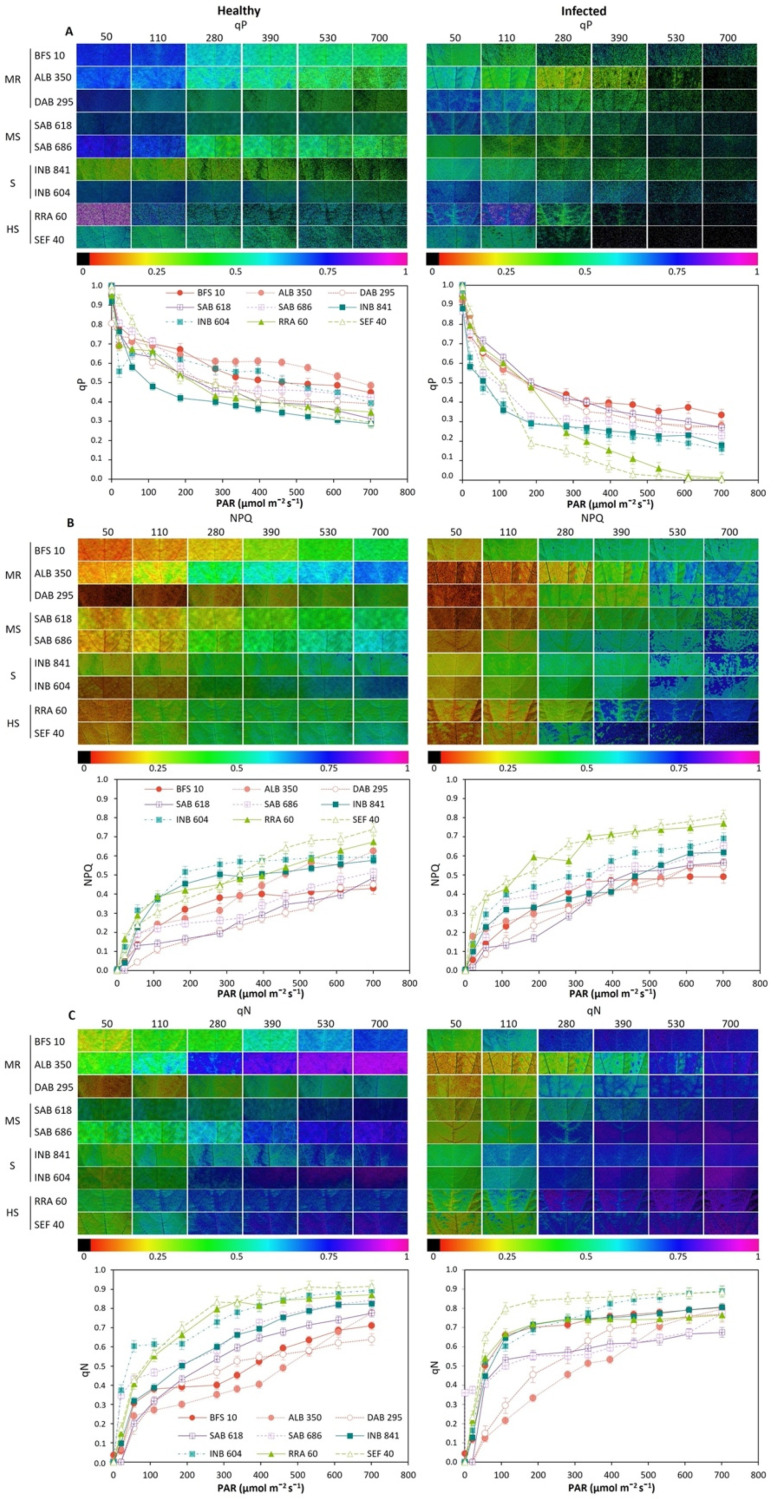
Response to disease stress as a function of the photosynthetically active radiation (PAR) level of common bean lines conforming different typologies. BSF 10, ALB 350, DAB 295: moderately resistant (MR). SAB 618, SAB 686: moderately susceptible (HS). INB 841 and INB 604: susceptible (S). RRA 60 and SEF 40: highly susceptible (HS). (**A**) The coefficient of photochemical quenching (qP); (**B**) non-photochemical quenching (NPQ); (**C**) non-photochemical cooling coefficient (qN). Images obtained with IMAGINE-PAM, the change in color gradient from violet to red signifies a change in value from higher to lower for each variable. Means and error bars correspond to 96 AOIs (areas of interest) for each condition (healthy and infected); i.e., six AOIs were taken for each leaf, among four plant leaves selected between the fifth and eighth trifoliate leaves (from apex to base) at the beginning of physiological maturity, from four plants of each advanced line of common bean.

For the photochemical quenching coefficient (qP) level, a decrease in the color gradient from blue to light green was found. This resulted in a decrease in qP with increasing PAR (Figure 8A). Among the advanced bean lines, ALB 350 had the highest fraction of energy devoted to photochemical quenching (qP, *p* < 0.05, Figure 8A) in the healthy leaf. BFS 10 showed the highest qP values in infected leaves during the rapid light curve (Figure 8A). Therefore, those lines that reduced their energy fraction in qP increased the dissipation of energy in the form of heat (NPQ, Figure 8B) or non-regulated processes (qN, Figure 8C). Thus, advanced lines of the highly susceptible typology such as SEF 40 and RRA 60 presented the highest values. In these cases, the color gradients for NPQ (Figure 8B) in the healthy leaves tended towards green while in the infected leaves the NPQ value increased as the color gradient changed from blue to violet from the level of 300 μmol m^−2^ s^−1^ of PAR, and a similar condition was observed for qN.

### 2.4. Correlations of Disease Susceptibility Index (DSI) with Chlorophyll A (Chl_a_) Fluorescence Parameters and Agronomic Variables

Chlorophyll a (Chl_a_) fluorescence parameters were found to be related to the agronomic variables that were also related to DSI (Table 2). For example, DSI was negatively correlated with most parameters of chlorophyll fluorescence, significantly affecting the fraction of energy devoted to the photosynthetic process (qP ratio coefficient r = −0.82) as well as the rate of electron transfer (ETR r = −0.72). Increasing the susceptibility level increased photosynthetic efficiency (r = 0.27) as well as leaf temperature (r = 0.32), but the difference in leaf temperature (leaf temperature differential, LTD) had a negative relationship with the photosynthetic efficiency (r = −0.29). With the dry matter accumulation variables, DSI was positively correlated with the increase in root biomass (r = 0.28), but it was negatively correlated with the other parameters such as stem biomass (r = −0.4), pod biomass (r = −0.29) and canopy biomass (r = −0.33). When leaf temperature increased, the functioning of the photosynthetic apparatus was compromised, specifically for photosynthesis (qP r = −0.3) and the rate of electron transfer (ETR r = −0.37). However, when leaf temperature increased, the plant also increased the dissipation of energy in the form of non-regulated energy (YNO, r = 0.36). At the agronomic level, the increase in leaf temperature compromised the number of grains per pod (r = −0.43) because the number of aborted grains increased too (r = 0.36). Likewise, leaf temperature correlated negatively with the chlorophyll content index (CCI r = −0.41), as well as with ambient temperature (r = −0.9), relative humidity (r = −0.52) and leaf temperature differential (r = −0.52). Since we observed a negative relationship between different parameters of chlorophyll a (Chla) fluorescence (ETR, qP and NPQ) and DSI, other important correlations were explored to improve confidence in the phenotyping of the advanced bean lines. For example, significant correlations were found between the ETR and stem biomass (r = 0.36) and the fraction of energy devoted to photosynthesis (qP) and pod biomass (r = 0.30). Finally, the energy dissipated as heat (NPQ) was negatively correlated with root biomass (r = −0.31).

## 3. Discussion

### 3.1. Magnitude of Leaf Disease Severity Modifies Energy Pathways within the Photosynthetic Apparatus

In our study we found that pathogen-related biotic stress generated differential responses in chlorophyll fluorescence parameters. The changes related to the increase of initial fluorescence (F_0_), the reduction of maximum fluorescence (F_m_) and the maximum efficiency of PSII photochemistry (F_v_/F_m_). These parameters responded by activating acclimation mechanisms with the aim of adjusting the photosynthetic machinery to the new conditions [40]. The interaction between plant and pathogen caused alterations in several physiological processes, in which photosynthetic reaction to light, carbon assimilation and respiration-associated processes were negatively affected [41]. We found that the maximum efficiency of PSII photochemistry, determined by F_v_/F_m_, decreased to values below 0.80 in disease-stressed leaves [42], and this could be considered as a response to the infection process presenting greater damage to the photosynthetic apparatus, resulting in two different processes [43]. On the one hand, the decrease in F_v_/F_m_ revealed that infection interferes with chloroplast protein synthesis, confirming that oxidation causes detrimental effects at the chloroplast level [44]. On the other hand, the decrease in F_v_/F_m_ may be attributed to changes in the number of functional reaction centers of PSII, presenting increased inactivation of reaction centers through damaging processes, and/or an increase in the rate constant of the non-radiative dissipation of excitation energy [13,14]. Likewise, the decrease in the rate constant of the PSII photochemistry led to an increase in the initial fluorescence at the PSII open centers (F_0_), a feature that was observed in infected leaves that showed increases in F_0_ compared to healthy leaves. This suggests a loss of excitation energy, which is required during electron transfer from pigments to reaction centers [45]. In the case of reduced maximum fluorescence (F_m_) in infected leaves, it is possible that maximum quinone reduction did not occur, because reaction centers may not function properly due to an operationally inefficient light energy capture process [19,46].

We found that infected leaves had to make certain adjustments to cope with the infection process, such as decreases in Y(II) and qP photochemical quenching, resulting in a decreasing electron transport rate (ETR) compared to healthy leaves [40]. Moreover, this inhibition of light-dependent pathways was accompanied by an increase in qN [47]. Similarly, an increase in Y(NO) was detected at the expense of a decrease in NPQ condition, as was also observed by Suárez at el. [48]. These observed changes are possibly attributable to irreversible damage in the PSII reaction centers due to the infection process, and they resulted in possible photoinhibition as reflected by low F_v_/F_m_ levels [49]. However, greater energy allocation towards the heat dissipation or non-photochemical pathway (NPQ) was observed in healthy leaves compared to infected leaves. This could indicate that pathogen infection may increase or decrease non-photochemical processes of energy dissipation [40,49].

Looking specifically at the behavior of NPQ we found certain adjustments due to the incidence of infection. It would be expected that greater allocation of energy to the non-photochemical pathway (NPQ) is presented as a photoprotective mechanism [4]. This would likely lead to lower stomatal conductance and increasing photosynthetic function, thus limiting the negative feedback on biomass productivity and/or acting as an escape from disease stress [50]. However, we observed that in infected leaves, the energy allocation to NPQ was lower than that presented by healthy leaves. This situation can lead to a low pH gradient (ΔpH) across thylakoid membranes, reflecting a high ATP demand due to metabolic stimulation in the host plant leaves [49]. Likewise, it has been observed that these decreases in NPQ denote a loss of chloroplast functionality, possibly related to leaf senescence [51]. Furthermore, under infected conditions, we found negative correlations between Y(NO) and the non-photochemical energy pathways of NPQ, qN and Y(NPQ), and low correlations between F_0_, Y(II), qP and qL. The above-mentioned relationships indicate that infected leaves devoted a larger proportion of the energy absorbed by PSII to other non-regulated energy dissipation processes [Y(NO)] [52]. The increase in Y(NO) reflect the plant’s inability to protect itself against photochemical damage [53]; i.e., high Y(NO) values and low NPQ values are reflected in a suboptimal capacity for photoprotective reactions, which ultimately leads to photoinhibition [52].

### 3.2. Physiological Response Differences among Common Bean Lines Reflect Possible Disease Resistance Mechanisms

Plant defense responses in terms of photosynthetic activity have mostly been studied independently in the past [54]. However, it has been observed that it is possible to identify materials that are disease-resistant in addition to serving as a basis for signal transduction for plant immune defense [55,56]. The common bean originates from two main gene pools and some diseases [8], due to the process of coevolution, present the same classification and higher degrees of pathogenicity within specific gene pools [9,57]. These studies indicate that plant photosynthesis and immune defense processes are interconnected [58]. In our study, climatic conditions (high air humidity, high rainfall and high air temperature) in the Amazon region were favorable for the more frequent development of diseases. However, in the previous studies conducted by Suárez et al. [48,59,60], we identified bean lines that were adapted to abiotic stress conditions at the physiological level and these lines produced grain yields that were above the Colombian average yield.

The specific adaptation level of a bean line depends on its physiological responses and degree of susceptibility to a given stress condition. For example, based on the physiological responses of the lines evaluated under healthy and infected conditions, we classified bean lines into four typologies. We found six common bean lines belonging to the moderately disease-resistant typology, with five of them belonging to the Mesoamerican gene pool (ALB 350, SMC 200, BFS 10, SER 16, SMN 27) and one belonging to the Andean gene pool (DAB 295), with a higher energy allocation towards photochemical pathways (Y(II), qP, qL, ETR) [61]. DAB 295 stands out as a line that was characterized by a greater allocation capacity in Y(II), allowing it to maintain higher ETR levels than the other bean lines. Likewise, its high qP and qL values indicated greater Q_A_ oxidation with a greater proportion of PSII reaction centers open and connected, showing optimal functioning in the photosynthetic apparatus [13]. In the case of the ALB 350 and BFS 10 lines, it was observed that they maintained a higher proportion of energy allocation to Y(II). However, these two bean lines belonging to the moderately resistant typology were the ones that most used the non-photochemical pathway (NPQ) for improving their stress tolerance response [40,49]. Thus, these two lines responded to disease stress by activating the acclimation mechanisms to adjust the photosynthetic machinery with the objective of maintaining their photosynthetic activity, which included the increase in energy dissipation capacity, and this was detected by the increase of the non-photochemical pathway without altering the yield of PSII Y(II) [40].

The bean lines that formed the moderately disease-susceptible typology (SMR 84, SMC 33, SMR 193, RRA 3, SMR 72, SIN 509-1, SMR 140, RRA 177, SMC 101, SMC 205, CALIMA, DAB 525, SMC 216, SMC 199, SAB 618 and SAB 686) had an average DSI value of 0.33, with a range from 0.24 to 0.39. This typology was characterized by lower efficiency in the functioning of the photosynthetic apparatus compared to the lines of the moderately resistant typology. Some outstanding traits were found in the old commercial Andean bean cultivar CALIMA, which presented higher NPQ and qN values [40], indicating higher dissipation of excess energy in PSII in the form of heat as a protection mechanism against disease stress [62,63]. This change in energy allocation allowed the CALIMA bean cultivar to maintain higher values in qP and qL with a high energy fraction (Y(II)), similar to that presented by the ALB 350 bean line, allowing photosynthetic activity to be maintained intact and without being affected by the rate of electron transfer to PSII [4,64]. The bean line SMC 33 exhibited high values of qP and therefore the infection from the pathogen did not generate damage to the reaction centers of PSII, as they remained open without affecting the reduction in the proportion of energy used for photochemical processes, which resulted in higher values of ETR [65].

The incidence of the pathogens did not favor the bean lines SER 394, ALB 352, ALB 351, ICA QUIMBAYA, SIN 351-1, SMC 135, RRA 60 and SEF 40, which conformed to the highly disease-susceptible typology due to their values of DSI being above 0.69. This typology presented low values of qP, and this parameter is considered useful to estimate the saturation level of PSII under stress conditions [66]. These low qP values were due to a degradation of photosynthetic pigments in leaves, in addition to high production of ethylene and reactive oxygen species (ROS), which affected the biochemical function of the chloroplast [20]. Likewise, an increase in the NPQ fraction was observed with the genotypes ICA QUIMBAYA, RRA 60 and SIN 351-1. This effect is considered as a photoprotective mechanism in these lines to mitigate infection [23]. However, this increase was probably more associated with the limitation of CO_2_ assimilation, possibly due to an imbalance between the activity of PSII and the electron requirement for photosynthesis, which was reduced due to disease stress, and the above process was supported by the low ETR values observed [67]. These conditions led to an overexcitation of light energy and subsequent photoinhibition [68]. Other lines, such as SEF 40, SMC 135, ALB 352 and ALB 351, only presented an increase in non-photochemical dissipation (qN), which was reflected through the activation of non-photochemical processes that led to the dissipation of non-radiative energy, leading to changes in the trans-thylakoid pH gradient and consequently to photoinhibition and disconnection among light-harvesting complexes [69].

### 3.3. Identification of Mechanisms of Energy Use among Moderately Resistant and Susceptible Bean Lines

Pathogen incidence is known to disrupt the function of the photosynthetic apparatus, resulting in differences among the bean lines [44]. Based on the response mechanisms observed among different bean typologies, we found two types of mechanistic responses: (i) greater allocation of energy to the photochemical pathway; and (ii) the ability to distribute energy to non-photochemical pathways. For example, in lines within the moderately susceptible (SAB 618, SAB 686), susceptible (INB 841, INB 604) and highly susceptible (RRA 60, SEF 40) typologies, fluorescence imaging showed increased values in the maximum efficiency of PSII photochemistry in infected leaves compared to healthy leaves. This stress-induced decrease in the F_v_/F_m_ ratio may result in an increase in the non-photochemical quenching processes, which decreases the value of F_m_ [70]. This can also be accompanied by photoinactivation, leading to oxidative damage and loss of PSII reaction centers [71], both of which are associated with increased value of F_0_ [72,73].

We found that the function of the photosynthetic apparatus is severely affected in infected leaves. For example, with the RRA 60 and SEF 40 lines, PSII Y(II) yield was proportionally reduced with increasing PAR level starting at 300 μmol m^−2^ s^−1^, indicating no photosynthetic activity at 500 μmol m^−2^ s^−1^ and resulting in total loss of electron transport at a PAR level of 600 μmol m^−2^ s^−1^. This behavior was expected because of the disease severity leading to inhibition of light-dependent reactions (Y(II), qP and ETR), and causing a severe loss of PSII function [40,47]. This loss was due to a reduction in the capture of excitation energy from open PSII reaction centers [74], which promotes an increase in the proportion of closed PSII reaction centers [67]. Thus, since the F_v_/F_m_ ratio represents the balance between the PSII photodamage rate and the PSII repair rate [71] and it is not related to changes in light absorption [75], we hypothesized that infection interferes with chloroplast protein synthesis [44].

### 3.4. Traits Associated with Lower Values of DSI Favor Better Agronomic Performance

When analyzing the agronomic performance of the evaluated bean lines we found that the lines that conformed to the moderately resistant typology (ALB 350, SMC 200, BFS 10, SER 16, SMN 27, DAB 295) had a greater capacity to increase biomass production at the canopy level. This was due to a greater resistance to disease stress (lower leaf damage) that made it possible to allocate more energy to the photochemical pathway (qP), contributing to a better rate of CO_2_ fixation and plant development. Thus—in similar environments with comparable soil conditions—higher CB values could be an indicator of a genotype’s greater capacity to fix CO_2_, assimilate nutrients and effectively use water under both stress conditions as well as optimal conditions.

The DSI was created with the objective of being a phenotyping tool for the selection of bean lines with lower susceptibility to disease stress based on Chl_a_ variables. The DSI is based on calculating the differences in the responses of different Chl_a_ variables between healthy and infected leaves. The DSI uses the different chlorophyll fluorescence parameters related to different energy pathways, the electron transfer rate and the different points on the photosynthetically active radiation (PAR) response curve, which show the functional status of the photosynthetic apparatus. Therefore, DSIs based on chlorophyll fluorescence parameters could be useful to identify bean lines with higher levels of adaptation to combined biotic and abiotic (acid soil and high temperatures) stress conditions. This observation is consistent with previous studies by Suárez et al. [48,59,60,76]. These previous studies showed that a few bean lines (ALB 350, SMC 200, BFS 10, SER 16, SMN 27, DAB 295) are capable not only of greater accumulation of photosynthates but also their mobilization to developing grains under stress conditions. Therefore, we can highlight, among others, the BFS 10 line, which demonstrated a high degree of adaptation under different types of abiotic stress. This is because it has a high capacity in the functioning of its photosynthetic apparatus, as we observed in this study, and it also has greater capacity to maintain its physiological processes under both biotic and abiotic stress conditions. Thus, BFS 10 could serve as a very good parent to develop multiple stress-adapted common bean lines.

It has been shown that the high temperatures present in the western Amazon region of Colombia impact the phenology and grain yield of common bean lines [59,76]. These conditions can also effect the plant–pathogen interaction and thereby influence genotypic differences in disease resistance [77]. Bean lines of the moderately resistant typology presented phenotypic variation and plasticity [78] at the level of their photosynthetic apparatus. These lines, in addition to increasing energy devoted to carbon fixation (qP), equally increased other dissipation pathways in the form of heat (NPQ) or non-regulated (qN) energy. These moderately resistant bean lines can surely be used as progenitors in bean breeding programs that aim to combine disease resistance with abiotic stress (acid soil and high temperature) resistance.

## 4. Materials and Methods

### 4.1. Experimental Site and Meteorological Conditions

**Figure 9:**
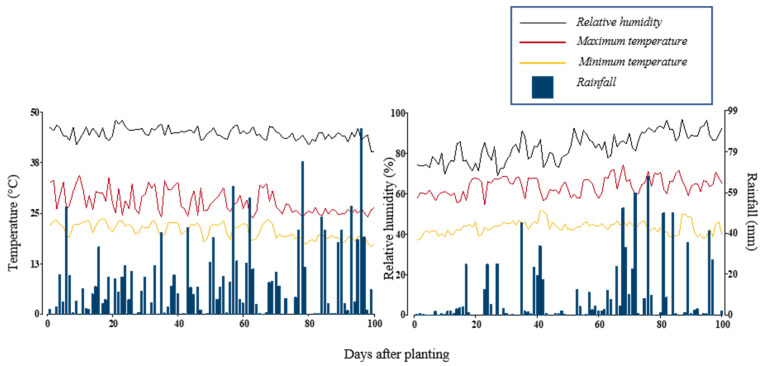
Distribution of rainfall and maximum/minimum temperatures during the crop growing period at the Macagual Research Center in Colombia during two seasons in 2019: April–July (**left**); and August–December (**right**).

A set of 59 bean genotypes were evaluated in two seasons ((i) April to July 2019, (ii) August to November 2019) at the Macagual Research Center of the Universidad de la Amazonia, Colombia (1°37′ N and 75°36′ W). The Center is located in the municipality of Florencia, Caquetá (Colombia), in a tropical rainforest ecosystem. The average annual rainfall is 3800 mm, with an average temperature of 25.5 °C and a relative humidity of 84% and 1700 h of sunshine per year. The first season had a mean temperature of 24.2 °C, with a minimum temperature range between 16.7–23.6 °C, maximum temperature between 23.9–34.4 °C and a total precipitation of 1,511 mm. The second season was characterized by a total precipitation of 844 mm and a mean temperature of 26.8 °C, with minimum temperatures ranging between 18.6–26 °C and maximum temperatures ranging between 27.2–37.2 °C (Figure 9).

### 4.2. Plant Material and Experimental Design

A total of 59 bean genotypes from the Mesoamerican and Andean gene pools from crosses between different species (*Phaseolus vulgaris*, *P. acutifolius*, *P. coccineus* and *P. dumosus*) were used (Supplementary Table S1). The ALB (small red kidney, black kidney) lines are better adapted to drought and acid soil conditions. BFS (small red) lines are adapted to drought and soils with low fertility. The DAB lines (speckled red, red-pink) are drought-adapted Andean materials selected for their color and grain size. The INB line is an interspecific progeny between the tepary bean and common bean, with some level of common bacterial blight (CBB) resistance. The RRA lines have outstanding performance in terms of resistance to root rot caused by *Pythium* and *Sclerotium*. The SAB line is heat and drought resistant and produces medium-sized cream-colored mottled seeds. The SAP line is of growth habit I with a mottled red grain color. The SEF and SER (small red) lines show adaptation to both drought and heat stress. The SIN (black) lines are interspecific with adaptation to drought. The SMC (various colors), SMN (black) and SMR (red) lines have high mineral content, specifically iron (Fe) in the seed, and are adapted to drought stress. The commercial cultivars ICA QUIMBAYA (cream mottled red) and CALIMA (red) are adapted to acid soil stress. AMADEUS (bright light red) is a commercial line. The experiment was conducted using a randomized complete block design (RCBD) with three replications where the plots in each block corresponded to each bean line evaluated. Each plot consisted of four rows with a distance between rows of 0.6 m, and each row was 2 m long. The bean seeds were sown at a distance of 15 cm, equivalent to 11 plants m^−2^. Soil preparation and planting were done manually. No amendments or products to regulate soil pH were added to the soil, nor was any type of chemical compound added to control the incidence of disease. The above-mentioned management ensures the similarity of the infection level conditions with those observed in the farmers’ fields in western Amazonia.

### 4.3. Selection of Infected and Healthy Leaves for Physiological Evaluation

**Figure 10:**
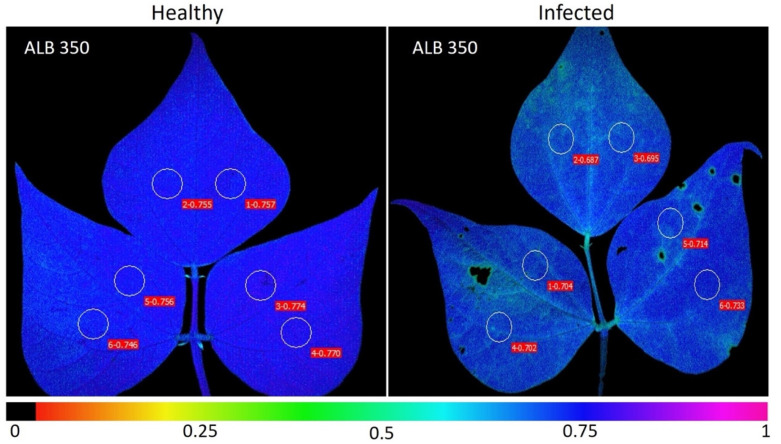
Selected areas of interest (AOIs) in each leaflet of healthy and infected leaves of *Phaseolus vulgaris*. The figure shows the maximum quantum efficiency of PSII (F_v_/F_m_) for healthy and infected leaves. The color gradient showing a blue color in healthy leaves indicates leaves without disease stress. The green color indicates a decrease in the value of the F_v_/F_m_ ratio as a response to disease stress.

During the flowering period (BBCH 65) in both crop-growing seasons under field conditions, eight plants per plot were selected in each block (n = 3). Four plants with healthy leaves (n = 4) located between the fifth and eighth trifoliate leaves (from the apex to the base) were selected as well as four plants with infected leaves (n = 4) with the presence of the asexual stage (*Rhizoctonia solani*) of the web blight (*Thanatephorus cucumeris* (Frank) Donk, [79]). The infected leaves showed the first visible symptoms with a severity level of four, which presented in the form of small wet spots that later turned into light brown spots with dark margins [12]. In both healthy and infected leaves, two circular areas of interest in the central part of each leaflet were selected for measurement (Figure 10). The area in which the information on different chlorophyll fluorescence variables was obtained (59 bean lines × 4 plants per line × 4 leaves per plant × 3 leaflets × 2 AOIs per leaflet, for a total of 5664 units of data per each of the healthy or infected leaf condition per block). This evaluation was carried out in the field to evaluate the disease by observing the natural occurrence of the web blight in the most appropriate stage, which corresponded to the main flowering period [80]. The objective of the mentioned management was to obtain better discrimination between the lines for their disease susceptibility by identifying a number of lines based on the disease susceptibility index (DSI) and finding the relationship of the DSI with the grain yield [81,82].

### 4.4. Chlorophyll (Chl_a_) Fluorescence and Imaging for the Chla Parameters

Responses of Chl_a_ parameters in healthy and infected leaves were monitored using a MAXI-Imaging PAM M-Series (Heinz Walz GmbH, Effeltrich, Germany), equipped with 44 high-power royal blue (450 nm) LED lamps furnished with collimating optics that provided pulse-modulated excitation light and at the same time served for actinic illumination and saturation pulses. For the calibration of the light intensities, an ULM-500 light meter and recorder was used, which was equipped with a micro quantum sensor (Heinz Walz GmbH, Effeltrich, Germany). Chlorophyll a (Chl_a_) fluorescence emissions were captured using a CCD (charge-coupled device) camera with a resolution of 640 × 480 pixels and a visible sample area of 24 × 32 mm on each leaf. From the color gradients and trends with increasing photosynthetically active radiation (PAR) obtained with the Imaging-PAM M-Series and Imaging WIN version 2.32 software (Heinz Walz GmbH, Effeltrich, Germany), different parameters related to chlorophyll fluorescence (Chl_a_) were determined. The bean leaves were individually fixed to a holder at a distance of 18.5 cm from the CCD camera in order to perform a 60-min dark adaptation process, a process that was performed with the aim of fully opening (i.e., oxidizing) the PSII reaction centers and minimizing non-photochemical energy dissipation.

After adaptation, the leaves were exposed to a weak, modulated light beam (0.5 μmol m^−2^ s^−1^, 100 μs, 1 Hz) with the aim of determining the initial fluorescence (F_0_). A saturating pulse of white light (2400 μmol m^−2^ s^−1^, 10 Hz) was then emitted for 0.8 s to determine the emission of maximal fluorescence (F_m_) or when all PSII reaction centers were expected to be “closed”. After 10 min, the steady-state chlorophyll fluorescence yield (F_s_) of plants under actinic illumination was determined and a second saturation pulse, with the same settings as for determining F_m_, was applied to measure the maximum light-adapted fluorescence yield (F_m_′). After the application of continuous actinic light saturating pulses, the fluorescence level increased to the F_m_ level and up to the F_m_′ level, thereby determining the amount of energy taken by the photochemical pathway [qP = (F_m_′ − F)/(F_m_′ − F_o_′)] [83]. Using the above protocol, different parameters of Chl_a_ in dark-adapted and light-adapted states were calculated, as described as follows. The maximum quantum yield of the PSII photochemistry was calculated (F_v_/F_m_ = (F_m_ − F_o_)/F_m_) as well as the different routes of the energy fractions. Among these were the effective quantum yield of photochemical energy conversion in PSII (Y(II) = ∆F/F_m_′) as well as the quantum yield of non-regulated non-photochemical energy loss in PSII (Y(NPQ) = (F_s_/F_m_′) − (F_s_/F_m_)) and the quantum yield of non-regulated heat dissipation and fluorescence emission (Y(NO) = F_s_/F_m_) [84]. The sum of these three yields is equal to 1.

In order to determine the incidence of the irradiance level on the PSII fluorescence emissions, rapid light curves (RLCs) composed of 12 steps, with durations at each PAR level of 20 s, were performed (0, 1, 21, 56, 111, 186, 281, 336, 396, 461, 531, 611, 701 µmol photons m^−2^ s^−1^). At each point on the curve, the electron transport rate (ETR) of the plants was calculated using the following formula: ETR (μmol e^−^ m^−2^ s^−1^) = (ΔF/F_m_′) × PAR × 0.5 × 0.84 [85,86] where ∆F/F_m_′ refers to the effective quantum yield of photochemical energy conversion into PSII [i.e., Y(II)] at a given radiation level; PAR is the nominal incident photon flux density in terms of µmol m^−2^ s^−1^; 0.5 refers to an assumed stoichiometric distribution of absorbed light energy between the two photosystems; and 0.84 denotes an empirical absorption efficiency of 84% of incident light in higher plants. Using the results of the RLCs, the maximum ETR (ETR_max_), the maximum saturating irradiance (E_k_) and the photosynthetic efficiency (α) were calculated. α was calculated as the initial slope of the curve at the first light passage and ETR_max_ was considered as the highest calculated ETR value of the curve (ETR = ETR_max_ × tanh (αETR × PAR/ETR_max_), where ETR is the relative electron transport rate mentioned above, ETR_max_ is the saturated ETR, tanh is the hyperbolic tangent function, αETR is the efficiency of the electron transport (initial slope of the ETR vs. irradiance curves) and PAR is the incident irradiance [87].

### 4.5. Physiological, Environmental and Agronomic Performance Variables

Between 7:00 to 9:00 am, on four fully developed leaves selected between the fifth and eighth trifoliate leaves (nodes were counted from apex to base) at the onset of physiological maturity of each plant (n = 4), the leaf chlorophyll content index (CCI) was measured using the SPAD502 (Minolta Camera Co., Osaka, Japan) chlorophyll meter. Leaf stomatal conductance (g_s_), leaf temperature (LT, °C) and relative humidity (RH %) were measured using a leaf porometer (Model SC-1, Decagon Devices, Inc. Pullman, WA, USA). The difference between leaf temperature and ambient temperature was used to calculate the leaf temperature differential (LTD) [48]. In each plot, a row segment of 0.5 m (equivalent to an area of 0.3 m^2^) was selected, and five plants were collected for destructive sampling, an activity that was carried out at the time of harvest (BBCH 89) in which the dry biomass of the different components of the plant (root, stems, leaves, flower buds, flowers, pods) was recorded as the total biomass of the canopy. The average number of viable and non-viable grains per pod was also measured for each bean genotype.

### 4.6. Developing and Validating the Disease Susceptibility Index

The disease susceptibility index (DSI) was developed to quantify the level of pathogen influence on photosynthetic functioning under stress and non-stress conditions. The DSI was adjusted for the disease stress intensity presented in each season. This index eliminates the effect of intra- and intergenotypic variability, showing the level of susceptibility of individual genotypes in the context of the population median [48]. To develop the DSI, as a first step, an initial standardization ([0–1]) of the data was performed using the value obtained from each genotype’s chlorophyll fluorescence variable (Chl_a_ V_i_) divided by the geometric value of the whole individual experiment (year repeat); i.e., Chl_a_ V_i [0–1]_= Y_j_/Ÿ, where Chl_a_ V_i_ = any of the monitored fluorescence ith variables, Y_j_ = mean value of the individual jth genotype, Ÿ = geometric mean of the whole experiment and [0–1] are standardized values between a range of 0 to 1. Once the data were standardized, two sub-indices were calculated. The first sub-index (SI_1i_) considered the performance of the photosynthetic apparatus of the bean lines in healthy leaves from the Chl_a_ V_i_; this was calculated from the difference between the genotype that had the highest value obtained (Max Chl_a_ V_i_, the more the better or the less the better) and the value of each jth genotype (SI_1_ = Max Chl_a_ V_i_ − Chl_a_ V_ij_). The second sub-index (SI_2i_) considered the difference between each healthy leaf (HL) and one infected (AL) by the pathogen (SI_2i_ = HL Chl_a_ V_i_ − AL Chl_a_ V_i_) in each jth genotype. After obtained the two sub-indices for each variable, we added the differences calculated and then added the score obtained for all variables. Thus, we developed and validated the DSI by having a greater difference within any genotype, in terms of photosynthetic characteristics, between the healthy leaf and the infected leaf with the pathogen. Where higher DSI values were observed with certain genotypes, they were considered as bean genotypes with greater susceptibility to disease stress (DSI [0–1] = ΣChl_a_ V_i_ SI_1i_ + Chl_a_ V_i_ SI_2i_).

### 4.7. Data Analysis

Using the different chlorophyll fluorescence variables, such as DSI and grain yield, a cluster analysis was performed to identify moderately resistant, moderately susceptible, susceptible and highly susceptible bean genotypes [12,88]. From these typologies, a principal component analysis (PCA) was used to identify the relationship of genotypes with chlorophyll fluorescence variables and to test the effect of disease stress on the physiological response in maintaining a higher photosynthetic efficiency, a situation that was tested by means of a Monte-Carlo permutation test. Statistical differences for each of the chlorophyll fluorescence variables were analyzed by fitting a linear mixed model (LMM). For this purpose, the fixed effect within the LMM was the bean genotype, and the blocks within the monitoring period (repeated measures) were included as random effects. Assumptions of normality and homogeneity of variance were evaluated using an exploratory analysis of residuals. Differences between genotypes were analyzed with Fisher’s post-hoc LSD test with a significance of α = 0.05. Pearson correlation analyses were performed for each of the chlorophyll fluorescence variables in both healthy and disease stressed leaves, as well as analyses of correlation between DSI, LT, ETR, qP and NPQ with different chlorophyll fluorescence, environmental and agronomic variables. The graphs were visualized in four quadrants plotting the corresponding means and statistical significance on each axis. LMMs were performed using the lme function of the nlme package, and cluster analysis, PCA and graphical outputs were performed in the packages ade4, ggplot2, factoextra and corrplot in R language software, version 3.4.4 [89] (R Development Core Team, 2019), using the interface in InfoStat [90].

## 5. Conclusions

The photosynthetic apparatus of the common bean was markedly affected by leaf disease stress as revealed by chlorophyll fluorescence parameters (initial F_0_ and maximum F_m_ fluorescence; the maximum quantum yield of the PSII photochemistry F_v_/F_m_; the fraction of energy devoted to photochemical processes qP; and electron transfer rate ETR). Based on the chlorophyll fluorescence responses of naturally infected and healthy leaves of 59 common bean lines, we classified the bean lines into four typologies ((i) moderately resistant, (ii) moderately susceptible, (iii) susceptible, and (iv) highly susceptible) based on the disease susceptibility index (DSI). Five bean lines from the Mesoamerican gene pool (ALB 350, SMC 200, BFS 10, SER 16 and SMN 27) and one line from the Andean gene pool (DAB 295) were identified as promising genotypes that can combine disease resistance with abiotic stress resistance. These lines allocated a higher proportion of energy to photochemical processes, which increased the rate of electron transfer. Based on the results from this study, we suggest that DSIs generated from chlorophyll fluorescence responses to pathogen infection could serve as a phenotyping tool in common bean breeding programs for evaluating large number of genotypes under field conditions and for identifying and/or developing common bean lines with multiple stress resistances.

## Figures and Tables

**Table 1:** Chlorophyll (Chl_a_) fluorescence and physiological, environmental and agronomic variables of different types of common bean genotypes, classified into four groups based on the disease susceptibility index (DSI) when grown under acid soil and high temperature stress conditions in the field over two seasons.

Variable	Moderately Resistant	Moderately Susceptible	Susceptible	Highly Susceptible	*p*-Value
Disease susceptibility index (DSI)	0.16 ± 0.02 ^a^	0.33 ± 0.01 ^b^	0.48 ± 0.01 ^c^	0.69 ± 0.02 ^d^	<0.0001
Initial fluorescence (F_0_)	0.02 ± 0.01 ^a^	0.02 ± 0.01 ^a^	0.02 ± 0.01 ^a^	0.01 ± 0.01 ^b^	0.0028
Maximum fluorescence (F_m_)	0.04 ± 0.01 ^a^	0.04 ± 0.01 ^a^	0.03 ± 0.01 ^b^	0.02 ± 0.01 ^c^	<0.0001
Effective quantum yield of photochemical energy conversion in PSII (Y(II))	0.15 ± 0.02 ^a^	0.12 ± 0.01 ^a^	0.09 ± 0.01 ^b^	0.08 ± 0.02 ^b^	0.0008
Quantum yield of regulated energy dissipation (Y(NPQ))	0.13 ± 0.02 ^b^	0.15 ± 0.01 ^b^	0.19 ± 0.01 ^a^	0.21 ± 0.02 ^a^	0.0003
Quantum yield of non-regulated energy dissipation (Y(NO))	0.13 ± 0.01	0.13 ± 0.01	0.12 ± 0.01	0.12 ± 0.02	
Non-photochemical quenching (NPQ)	0.25 ± 0.02	0.23 ± 0.01	0.21 ± 0.01	0.2 ± 0.02	
Non-photochemical quenching coefficient (qN)	0.25 ± 0.03 ^b^	0.28 ± 0.02 ^b^	0.35 ± 0.01 ^a^	0.36 ± 0.04 ^a^	0.0004
Coefficient of photochemical quenching (qP)	0.29 ± 0.02 ^a^	0.23 ± 0.01 ^b^	0.16 ± 0.01 ^c^	0.09 ± 0.01 ^d^	<0.0001
Coefficient of photochemical quenching (qL)	0.17 ± 0.02 ^a^	0.14 ± 0.01 ^b^	0.12 ± 0.01 ^b^	0.07 ± 0.01 ^c^	<0.0001
Electron transport rate (ETR; μmol e^−^ m^−2^ s^−1^)	10.26 ± 0.93 ^a^	8.08 ± 0.65 ^a^	4.8 ± 0.41 ^b^	3.07 ± 0.67 ^b^	<0.0001
ETR_max_ (ETR maximum; μmol e^−^ m^−2^ s^−1^)	65.66 ± 9.59 ^a^	49.40 ± 6.18 ^b^	46.84 ± 5.33 ^b^	19.71 ± 4.18 ^c^	<0.0001
Maximum saturating irradiance (E_k_; μmol m^−2^ s^−1^)	458.1 ± 22.47 ^a^	365.31 ± 17.747 ^b^	358.17 ± 18.8 ^b^	163 ± 12.82 ^c^	<0.0001
Dark respiration rates (*R*_d_; μmol CO_2_ m^−2^ s^−1^)	7.49 ± 0.02 ^c^	9.6 ± 0.29 ^b^	10.27 ± 0.18 ^b^	23 ± 0.98 ^a^	<0.0001
Root biomass (g)	1.05 ± 0.24 ^a^	2.1 ± 0.38 ^b^	1.4 ± 0.25 ^a^	1.35 ± 0.64 ^a^	<0.0001
Stem biomass (g)	8.45 ± 0.12 ^a^	7.12 ± 0.94 ^a^	5.35 ± 0.7 ^b^	3.48 ± 0.48 ^c^	<0.0001
Leaf biomass (g)	8.62 ± 0.29 ^a^	9.26 ± 0.61 ^a^	7.8 ± 0.23 ^c^	5.87 ± 0.15 ^c^	<0.0001
Flower bud biomass (g)	0.01 ± 0.01	0.04 ± 0.01	0.01 ± 0.01		
Flower biomass (g)	0.04 ± 0.04	0.34 ± 0.13	0.05 ± 0.02	0.1 ± 0.10	
Pods biomass (g)	4.6 ± 0.29 ^a^	3.36 ± 0.45 ^b^	0.72 ± 0.44 ^c^	0.34 ± 0.22 ^c^	<0.0001
Canopy biomass (CB, g)	22.98 ± 2.01 ^a^	19.75 ± 2.38 ^a^	15.22 ± 1.93 ^b^	10.9 ± 1.25 ^c^	<0.0001

Mean ± standard error. a, b, c: Averages with a letter in common between rows were not significantly different at 5% probability.

**Table 2:** Correlation coefficients (r) between the disease susceptibility index (DSI), leaf temperature (L_T_; °C), electron transport rate (ETR; μmol e^−^ m^−2^ s^−1^), coefficient of photochemical quenching (qP), non-photochemical quenching (NPQ) and other plant attributes for 52 bean lines grown under acid soil and high temperature stress conditions.

Variables	Variables				
	DSI	L_T_	ETR	qP	NPQ
Disease susceptibility index (DSI)		0.32 *	−0.72 ***	−0.82 ***	−0.38 ***
Initial fluorescence (F_0_)	−0.49 ***	−0.19	0.06	0	0.04
Maximum fluorescence (F_m_)	−0.66 ***	−0.39 **	0.39 ***	0.27 *	0.1
Effective quantum yield of photochemical energy conversion in PSII (Y(II))	−0.52 ***	−0.42 ***	0.8 ***	0.67 ***	0.3 **
Quantum yield of regulated energy dissipation (Y(NPQ))	0.53 ***	0.07	−0.69 ***	−0.48 ***	−0.88 ***
Quantum yield of non-regulated energy dissipation (Y(NO))	−0.04	0.36 **	−0.19	−0.18	0.69 ***
Non-photochemical quenching (NPQ)	−0.38 ***	0.1	0.41 ***	0.23 *	
Non-photochemical cooling coefficient (qN)	0.48 ***	0.2	−0.65 ***	−0.36 **	−0.77 ***
Coefficient of photochemical quenching (qP)	−0.82 ***	−0.3 **	0.78 ***		0.32 **
Coefficient of photochemical quenching (qL)	−0.57 ***	−0.09	0.32 **	0.8 ***	0.1
Electron transport rate (ETR, μmol e^−^ m^−2^ s^−1^)	−0.72 ***	−0.37 **		0.77 ***	0.41 ***
ETR_max_ (ETR maximum; μmol e^−^ m^−2^ s^−1^)	−0.13	−0.09	0.31 *	0.29 *	0.07
Maximum saturating irradiance (E_k_; μmol m^−2^ s^−1^)	−0.15	0.04	0.31 *	0.12	0.08
Dark respiration rates (*R*_d_; μmol CO_2_ m^−2^ s^−1^)	−0.22	−0.05	0.01	0.17	−0.02
Light compensation point (*LCP*; μmol m^−2^ s^−1^)	−0.15	−0.06	−0.02	0.08	−0.01
Photosynthetic efficiency (α; μmol CO_2_ μmol protons^−1^)	0.27 *	−0.03	−0.33 **	−0.12	−0.22
Chlorophyll content index (CCI; SPAD)	0.13	−0.41 **	−0.13	−0.15	−0.09
Leaf temperature (L_T_; °C)	0.32 *		−0.37 **	−0.38 **	0.1
Stomatal conductance (g_s_; μmol H_2_O m^−2^ s^−1^)	−0.13	−0.23	0.17	−0.03	0.01
Ambient temperature (°C)	−0.19	−0.9 ***	0.27 *	0.07	−0.09
Relative humidity (%)	−0.07	−0.52 ***	0.14	−0.1	0
Leaf temperature differential (LTD; °C)	−0.29 *	−0.98 ***	0.35 **	0.19	−0.08
Root biomass (g)	0.28 *	−0.1	−0.13	0.05	−0.31 *
Stem biomass (g)	−0.4 ***	−0.15	0.36 **	0.22	0
Leaf biomass (g)	−0.2	0.04	0.2	0.03	0.04
Flower bud biomass (g)	−0.19	−0.07	0.17	0.15	0.15
Flower biomass (g)	−0.1	0.07	0.01	−0.01	0
Pod biomass (g)	−0.29 *	−0.11	0.23	0.3 **	0.11
Viable seeds (number)	−0.03	−0.43 *	0.27	0.01	−0.01
Non-viable seeds (number)	−0.03	0.36 *	−0.02	−0.06	0.15
Canopy biomass (CB, g)	−0.33 **	−0.11	0.32 *	0.15	−0.01

Mean values were used in the correlation analysis and *, ** and *** represent levels of significance at 0.05, 0.01 and 0.001, respectively.

## Data Availability

Data are available from the authors upon request.

## Supplementary Materials

The following supporting information can be downloaded at: https://www.mdpi.com/article/10.3390/plants11101371/s1. Supplementary Table S1: 59 bean genotypes from the Mesoamerican and Andean gene pools from crosses between different species (*Phaseolus vulgaris*, *P. acutifolius*, *P. coccineus* and *P. dumosus*).
